# Modification of the Marmarou model in developing countries

**DOI:** 10.1016/j.amsu.2020.07.029

**Published:** 2020-07-22

**Authors:** Rizha Anshori Nasution, Andi Assadul Islam, Mochammad Hatta, Prihantono, Cahyono Kaelan, Jeni Poniman, Harakati Wangi

**Affiliations:** a Department of Neurosurgery, Pelamonia Hospital, Makassar, Indonesia; b Doctoral Program of Biomedical Sciences, Faculty of Medicine, Hasanuddin University, Makassar, Indonesia; c Department of Neurosurgery, Faculty of Medicine, Hasanuddin University, Makassar, Indonesia; d Clinical Microbiologist Program, Faculty of Medicine, Hasanuddin University, Makassar, Indonesia; e Department of Surgery Faculty of Medicine, Hasanuddin University, Makassar, Indonesia; f Department of Pathological Anatomy, Faculty of Medicine, Hasanuddin University, Makassar, Indonesia; g Department of Internal Medicine, Pelamonia Hospital, Makassar, Indonesia

**Keywords:** Traumatic brain injury, Marmarou model, Modification, Limited facilities

## Abstract

**Introduction:**

Head injury is an injury or wound of the brain tissue due to external forces; it can cause a decrease or change in the status of consciousness. Many head injury models have used mice as experimental animals; the Marmarou model is the most famous and the most widely-used diffuse brain injury model. In this study, we slightly modified the Marmarou model. The purpose of this study is to help researchers examining head injuries in mice, especially those in developing countries who have limited facilities and infrastructure.

**Methods:**

This experimental research uses animals models (*Rattus novergicus*, strain Sprague Dawley) that fit several criteria, including male, aged 10–12 weeks, and body weight of 200–300 g. This study involves a slight modification on the tube used, with a 20 cm-long weight of 20 g. The blood samples for the following assays of ELISA and brain tissue samples were collected at 24 h and 4, 5, 6, and 7 days post-trauma.

**Results:**

A significant effect on the brain was seen with the Marmarou model modification, at a mass weight of 20 g and height of 20 cm, with 0.04 J energy produced. Changes were also seen in the histological features of brain tissue and the serum levels of AQP-4, F2 IsoPs, MPO, and VEGF from 24 h until 7 days after trauma.

**Conclusion:**

This report describes the development of an experimental head injury approach modifying the Marmarou model that is able to produce a diffuse brain injury model in mice.

## Highlights

• Head injury is an injury of the brain tissue due to external forces.
• The Marmarou model is the most famous and widely-used diffuse brain injury model in mice.
• A significant effect on the brain was seen with our modified Marmarou trauma model.

## 1 Introduction

Traumatic Brain Injury (TBI) is a major medical and socioeconomic problem worldwide, due to its high disability rate in people below 35 years of age and its place as a main cause of death in children and young adults [1]. In addition, poor public awareness has resulted in approximately 52,000 deaths and 80,000 disabilities among road users and motorists, due to high rates of brain injury caused by accidents [2].

TBI triggers several pathological processes in the brain shortly after trauma. This causes a direct mechanical disruption of brain tissue, along with an indirect mechanism [3] due to an acute inflammatory response, including: damage to the blood-brain barrier (BBB) [4]; edema formation; peripheral blood cell infiltration and activation of cell immunocompetence; and intrathecal release from many inflammatory mediators, such as interleukins and chemotactic factors [3,5]. Various mediators involved in forming or exacerbating these pathological processes have been identified [5], including VEGF (vascular endothelial growth factor), arachidonic acid, bradykinin [6], Ca2+, glutamate, and free radical oxygen, among others [3,[5], [6], [7]].

Adequate facilities and infrastructure are one of the important factors in planning and conducting good scientific research; however, sometimes the limitations of facilities and the infrastructure hinder a researcher in planning their research. The unavailability of complete laboratory animal facilities is an inhibiting factor to conducting quality research. Consequently, we designed a head trauma research model that can be applied to simple research laboratories, who can then contribute to research on head trauma in developing countries.

This study demonstrates a head trauma model with slight modifications to Marmarou's head injury model [8], with the aim that it should be easy to implement for researchers with limited or minimal equipment, facilities, and infrastructures, so that such research can be carried out in non-elite educational institutions and in basic research institutions in developing countries without the high costs of the traditional model.

## 2 Material and method

### 2.1 Animal

Five *Rattus novergicus* (strain: Sprague Dawley) male rats weighing 200–300 g were used in this study. All animal procedures received approval from the local Ethics Commission, under Number: 771/UN4.6.4.5.31/PP36/2019. The work was also carried out in line with the ARRIVE Guidelines for reporting animal research [9].

### 2.2 Sample checking

Blood and brain tissue samples were collected 24 h, 4 days, 5 days, 6 days, and 7 after treatment. Blood samples were examined by the ELISA Sandwich method and included aquaporin-4 (AQP4) (Catalog No. LS-F4077), myeloperoxidase (MPO), Catalog No. LS-F24875, and VEGF Sandwich ELISA (Catalog No. LS-F978) purchased from Life Span Biosciences Inc., along with mouse 8-iso-PGF2 from Mybiosource.com. (Category No. MBS7606827).

Brain tissue samples were taken at 24 h after trauma, then on days 4, 5, 6, and 7. Brain tissue processing was carried out through the paraffin and Hematoxylin Eosin (HE) staining methods.

### 2.3 Statistical analysis

The data were processed using Excel 2013 and SPSS version 23 (IBM Corp. Released 2015. IBM SPSS Statistics for Windows, Version 23.0. Armonk, NY: IBM Corp). Data on body weight and AQP-4, F2 IsoPs, MPO, and VEGF levels are presented in descriptive form.

### 2.4 Surgical preparation

The five male Sprague-Dawley mice—free of viruses and weighing 280–300 g—were maintained and fed with BR-1 feed without any other food for approximately 2 months before the experiment. The food was regularly given every day, with Aqua Dest as the drink. The cage was a standard-shaped cage and was routinely cleaned to support hygiene; it was given a 12-h dark/12-h light cycle, with controlled temperature and humidity.

### 2.5 Anesthesia procedures

**Fig. 1:**
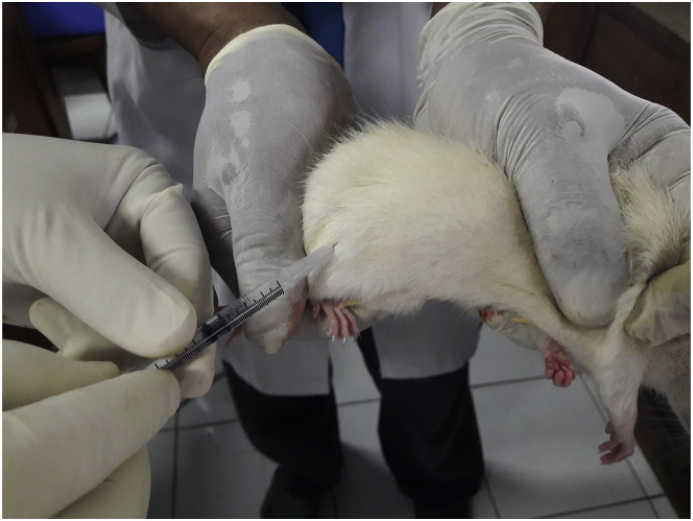
The anesthesia process for experimental animals.

Anesthesia was induced using the ketamine HCl diluted with 20 cc aqua distillate at a dose of 3–10 mg/kg, which was intramuscularly injected in the femoral region using an insulin syringe. Fig. 1 shows the process, which relied on continuation with slow aspiration to prevent the anesthetic from entering the blood vessels.

**Fig. 2:**
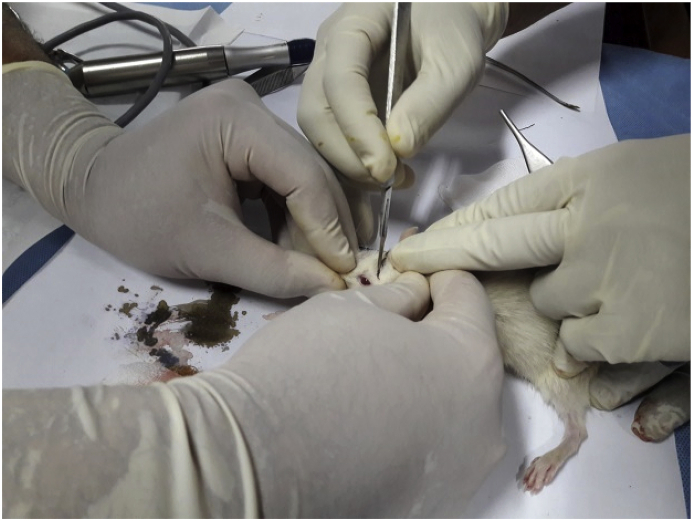
Surgical process.

**Fig. 3:**
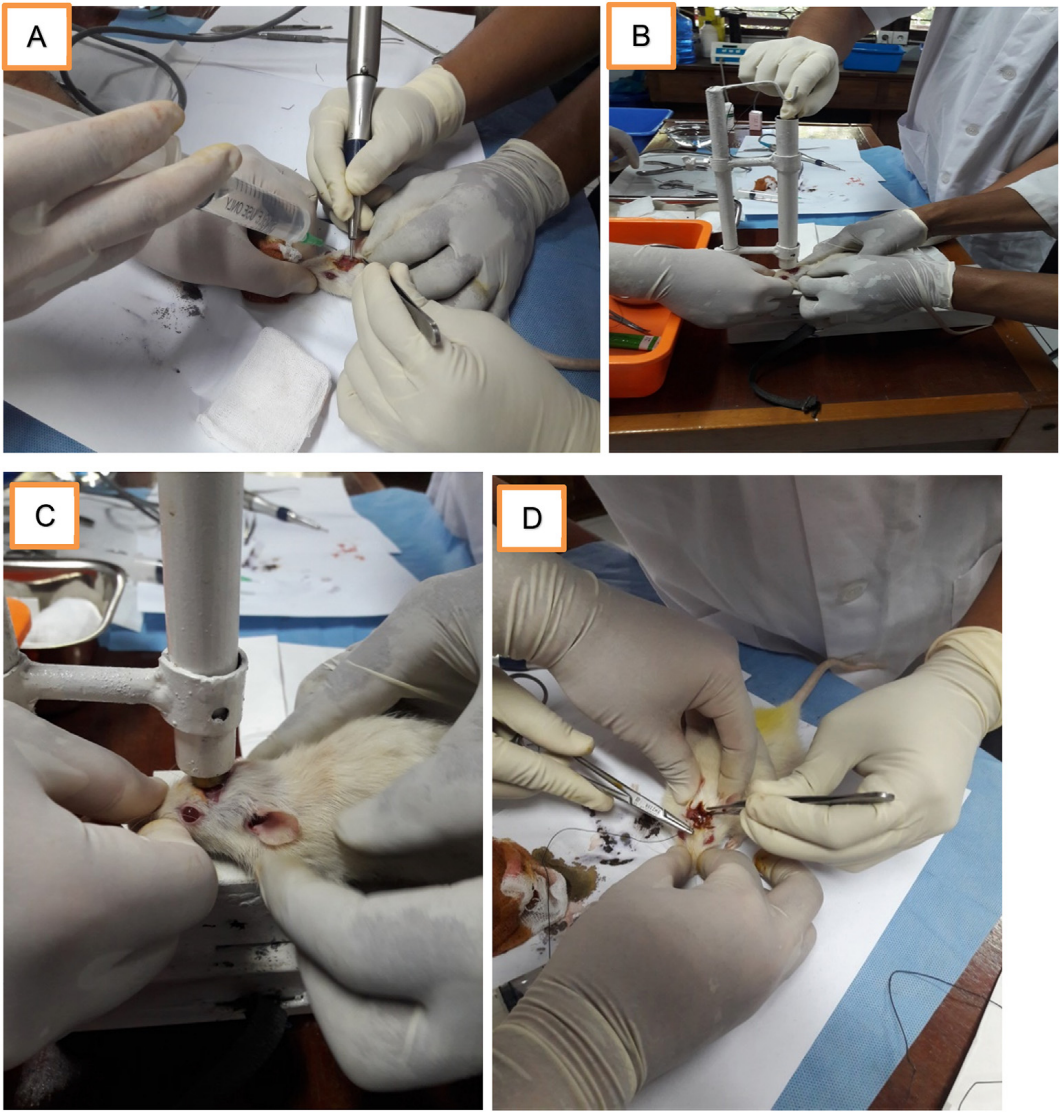
The treatment process of head trauma for experimental animals. A) A burr hole is created. B) The mouse head is placed under the tube. C). A mass of 20 g is dropped. D) Suturing.

### 2.6 Surgical procedure

- A linear incision was cut with no. 15 surgical blades through the midline of the mouse's skull at approximately 1–1.5 cm behind the coronary suture (Fig. 2). The incision was gently deepened with the *scalpel* to the periosteum and was further separated at the periosteum with a small Epson/dissector in order to locate the calvaria.
- A burr hole was created with a low-speed rill to open the calvaria bone layers in the external and internal tabula, thereby further exposing the dura. At that point, a small tang was used to generate a mini craniectomy measuring 1–1.5 cm in diameter (Fig. 3A).
- The mouse's head was placed just below the tube (Fig. 3B).
- A mass of 20 g was shot from inside the tube to hit the mouse's head (Fig. 3C).
- The wound was stitched with a simple suture and treated with topical antibiotics (Fig. 3D).

## 3 Results

**Fig. 4:**
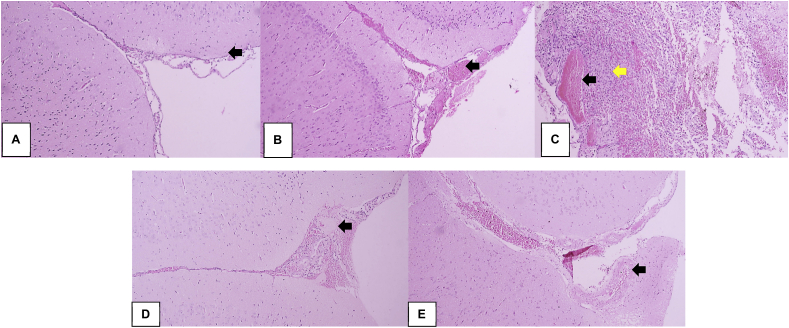
Histopathological examination of mice brain tissue (hematoxylin and eosin stain 40x). A) 24 h after trauma: brain tissue with minimal focus on blood vessel proliferation (black arrow). B) Day 4 after trauma: brain tissue with focus on blood vessel proliferation and dilatation (black arrow), as well as erythrocyte extravasation in the vicinity. C) Day 5 after trauma: brain tissue consisting of the glial cells' proliferation with a larger nucleus (glial reactive) (yellow arrow), accompanied by blood vessel proliferation and dilatation (black arrow) as well as erythrocyte extravasation in the vicinity. D) and E) Days 6 and 7 after trauma: brain tissue with focus on blood vessel proliferation and dilatation (black arrow), with emphasis on surrounding bleeding. (For interpretation of the references to colour in this figure legend, the reader is referred to the Web version of this article.)

The mice survived the trauma experiment, with a mean body weight of 290.07 g. Drawing on trauma models, experimental analysis on brain tissue damage showed the presence of minimal blood vessel proliferation on the first day; however, a continuous increase was reported up to the seventh day, as seen in the histological picture of brain tissues (Fig. 4).

**Fig. 5:**
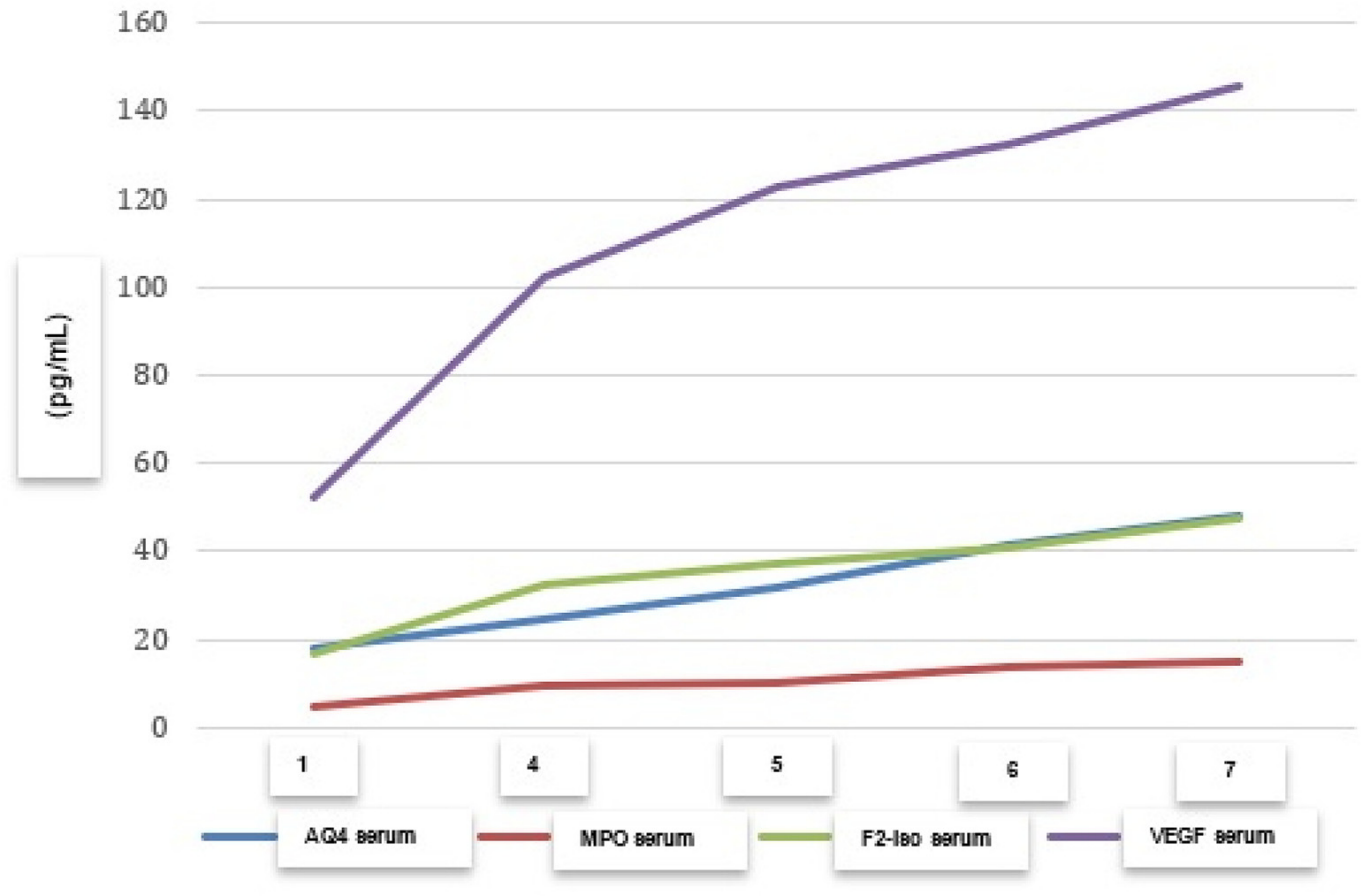
The parameters graph of AQP-4, MPO, F2-isoprostane, and VEGF based on time (days) after head injury.

The average levels of parameters AQP-4, MPO, F2-isoprostane, and VEGF showed a steady increase from days 1 through 4, 5, 6 and 7, as shown in Table 1 and Fig. 5.

**Table 1:** The Level of AQP-4, MPO, F2-isoprostane, and VEGF based on time (days) after head injury.

	AQ4 serum (pg/mL)	MPO serum (pg/mL)	F2-isoprostane serum (pg/mL)	VEGF serum (pg/ml)
24 h post trauma	18.03304	4.481512	17.09533	52.62576
Day 4	24.4322	9.329674	32.71747	102.2482
Day 5	31.8979	10.31446	37.52427	122.5483
Day 6	41.49664	14.02633	40.82896	132.3224
Day 7	47.89581	14.85961	47.43832	145.8558

## 4 Discussion

The Marmarou model is a well-known, widely-used diffuse brain injury standard [10] and is also called the "weight-drop" [11] due to the closed skull collision. This method is suitable for studying head trauma similar to falling or vehicle exhaustion cases, which are commonly experienced in hospital emergency departments.

The trauma model employed was a modification of previous work by Marmaorou in 1994 [12], using a 20-g weight with a height of 20 cm [13]. The load used is 6–10% of the total mouse body weight. In a conversion to human proportions [14], the load would be ± 3–5 kg, resulting in a large impact to the head [15].

In this model, the research uses a tube that is ±20 cm long and applies a physical formula to calculate the amount of energy at 0.04 J:

Total Mechanical energy (ME) = Potential Energy (EP) = Kinetic energy (EK) = m.g.h

with:

m = mass of body, 20 gr = 0.02 kg
h = height, 20 cm = 0.2 m
g = earth's gravity = 10 m/s [16,17]

The histological representation of the traumatized mouse brain tissue demonstrated a continuous daily increase in brain tissue proliferation and blood vessel dilatation, accompanied by erythrocyte extravasation in the vicinity. The results also showed an inflammation lasting from the first hour of the first day after the head injury. Additionally, there was evidence of secondary brain injury in the brain [[18], [19], [20]].

Besides tissue histology examination, several other factors related to head injuries were assessed, including AQP-4, MPO, F2-isoprostane, and VEGF. The aim was to investigate the cause-and-effect relationship between the trauma model used and the biochemical processes in the blood. Table 1 shows an increase in each indicator—evidenced by the chemical and inflammatory processes—recorded after the head injury [5]. These results also indicated a secondary brain injury process, due to the trauma model performed on the mice [21].

The increase in blood aquaporin is due to a damaged indicator induced by astrocytes and is key in cerebral edema [22]. The MPO test is widely used to measure the accumulation of polymorphonuclear leukocytes (PMNL) during myocardial infarction, lung infections, and skin and intestinal inflammation [23,24]. F2-IsoPs serve as a marker for accessing oxidative damage in vivo [25]. VEGF is known as a vascular permeability factor, resulting from the ability to induce vascular leakage; however, the unwanted negative response from the increasing values (in the form of expanded cerebrovascular permeability) leads to edema formation in the brain [26].

The Marmarou model modification—with a mass weight of 20 g, height of 20 cm, and energy production of 0.04 J—had a significant effect on the brain. This can be seen in changes in brain tissue histology and in the serum levels of AQP-4, F2 IsoPs, MPO, and VEGF from 24 h until 7 days after the trauma.

## 5 Conclusion

This report describes the development of a modification of the Marmarou model that is able to produce diffuse brain injury in mice. We modified the Marmarou trauma model by using a mass that weighs 20 g and is 20 cm tall; this can be used as a trauma model in head injury studies that use mice as experimental animals.

## Ethical approval

All procedure for Animal experiment has been approved by Ethics Commission Faculty of Medicine, Hasanuddin University, Number: 771/UN4.6.4.5.31/PP36/2019.

## Funding

No funding or sponsorship.

## Author contribution

RAN, AAI, MH, PRI, CK, JP, and HW wrote the manuscript and participated in the study design. RAN, AAI, CK, JP, and PRI drafted and revised the manuscript. RAN, AAI, and PRI performed head trauma treatment and surgery. AAI, MH, CK, JP, and PRI performed bioinformatics analyses and revised the manuscript. All authors read and approved the final manuscript.

## Registration of research studies

None.

## Guarantor

Rizha Anshori Nasution.

## Provenance and peer review

Not commissioned, externally peer reviewed.

## Declaration of competing interest

The authors declare that they have no conflict of interests.
